# miRNA-223 expression in patient-derived eutopic and ectopic endometrial stromal cells and its effect on epithelial-to-mesenchymal transition in endometriosis

**DOI:** 10.1016/j.clinsp.2022.100112

**Published:** 2022-10-14

**Authors:** Yuan Xue, Xueyan Lin, Tingting Shi, Yongjie Tian

**Affiliations:** a Department of Obstetrics and Gynecology, Shandong Provincial Hospital, Cheeloo College of Medicine, Shandong University, Shandong, China; b Department of Obstetrics and Gynecology, Shandong Provincial Hospital Affiliated to Shandong First Medical University, Shandong, China

**Keywords:** miRNA-223, Endometriosis, SCs, Epithelial-to-mesenchymal transition, Migration, Proliferation, Apoptosis

## Abstract

**Objective:**

This study was designed to evaluate the expression of microRNA-223 (miRNA-223) in patient-derived eutopic and ectopic endometrial stromal cells (SCs). Given the fact that miRNA-223 was previously shown to be upregulated in these cells and that this upregulation has been linked to epithelial-to-mesenchymal transition (EMT) during endometriosis, this study aimed to further explore the expression of miRNA-223, its effect in endometriosis, and the mechanisms underlying its effects.

**Methods:**

Endometrial tissue was collected from 26 patients with endometriosis and 14 patients with hysteromyoma (control group). Primary endometrial SCs were isolated and cultured from several endometrial samples and miRNA-223 expression was evaluated using qRT-PCR. Cells were then transfected with a miRNA-223 overexpression lentiviral vector (sh-miR-223 cells) or an empty control (sh-NC cells) and then used to monitor the effects of miRNA-223 on the expression of several EMT-associated proteins, including N-cadherin, vimentin, and Slug, using western blot. Cellular migration, invasion, and proliferation were then evaluated using a wound healing, Transwell, and CCK-8 assay, respectively. Flow cytometry was used to detect apoptosis.

**Results:**

There was a significant decrease in the expression of miRNA-223 in both eutopic and ectopic endometrial SCs (p < 0.05) whereas upregulation of miRNA-223 inhibited the expression of EMT-related molecules and reduced cell migration, invasion, and proliferation. High levels of miRNA-223 also promoted apoptosis.

**Conclusion:**

miRNA-223 expression decreased in endometrial SCs from endometriosis patients, which may facilitate the differential regulation of EMT during endometriosis.

**Clinical Trial registration number:**

SWYX2020-211.

## Highlights

• miRNA-223 was downregulated in endometrial stromal ells from endometriosis patients.
• miRNA-223 upregulation repressed malignant behaviors of endometrial stromal cells.
• miRNA-223 might serve as a potential therapeutic target for endometriosis.

## Introduction

Endometriosis is a condition of the female reproductive tract, which generally occurs in women of childbearing age. It is characterized by the presence of active endometrial tissue (glands and stroma) outside the womb. The incidence rate of endometriosis continues to increase every year and affects between 10% and 15% of the female population. Common symptoms include pelvic pain and infertility, which severely compromise the quality of life of patients.^1^^,^^2^

Endometriosis, defined as the presence of endometrial glandular and stromal cells outside the uterine cavity, is a common gynecological disease with poorly understood pathogenesis. Eutopic and ectopic stromal cells from patients with endometriosis exhibit differential invasive, adhesive, and proliferative behavior. Therefore, the characterization of the differences and similarities between the eutopic and ectopic endometrium is arguably a first important step toward the understanding of the pathogenesis of endometriosis. MicroRNAs (miRNAs) are endogenous non-coding RNAs with a length of 19–23 nucleotides, which regulate gene expression at the transcriptional or post-transcriptional level, participating in diverse cellular processes.^3^ Human miRNA-223 is located on the X chromosome, and its target genes are known to be involved in various biological processes, including signal transduction, transcriptional regulation, as well as cell growth and development. Recent studies evaluating the function of miRNA-223 reported that it modulates inflammation, infection, and cancer development.^4^ Further, miRNA-223 is known to be upregulated in the ectopic endometrium of patients with endometriosis when compared to that in the corresponding eutopic endometrium.^5^ In addition, patient-derived eutopic endometrium stromal cells (SCs) exhibited increased miRNA-223 expression compared to the surrounding epithelial cells.^6^ miRNA-223 was reported to be involved in Epithelial-to-Mesenchymal Transition (EMT) in various diseases. In cervical cancer, the expression of miRNA-223 was lower than in normal tissues, promoting EMT in HeLa cells.^7^ Studies have also confirmed the occurrence of EMT during endometriosis.^8^^,^^9^ Thus, miRNA-223 may be a key regulator of EMT during endometriosis. The aim of this study was to confirm miRNA-223 expression in SCs from patient samples and explore its role in EMT during endometriosis.

## Materials and methods

### Clinical specimens

Endometrial samples were collected from 40 patients who were hospitalized at the Shandong Provincial Hospital, Cheeloo College of Medicine, Shandong University between August 2019 and October 2020. Eutopic SCs (EuSCs, 16 cases) and ectopic SCs (EcSCs, 10 cases) were obtained from 26 patients with endometriosis. Control cells were obtained from the 14 remaining patients diagnosed with hysteromyoma. All the samples used in this study were evaluated and confirmed by a surgical pathologist. The following patients were included: females who were of childbearing age; had a regular menstrual history; had no history of sex hormone-related diseases except for endometriosis; had no malignant tumors; did not receive sex hormone-related drugs for at least 6 months prior to surgery; and agreed to the use of their tissue samples for experimental research. The mean age of the patients was 41.91±7.90 years (range, 23–55 years). This study was approved by the Ethics Committee at the Shandong Provincial Hospital, Cheeloo College of Medicine, Shandong University (protocol number SWYX2020-211). All study participants provided written informed consent before participating in the study.

### Major materials and reagents

The Dulbecco's Modified Eagle's Medium (DMEM)/F12 medium and collagenase used in the isolation and culture of the primary endometrial cells were procured from Sigma (USA). Fetal bovine serum was produced by BI (Israel) and the lentivirus used for transfection was packaged by Shanghai Jiman Technology Co., Ltd. The RIPA lysate buffer and the Bicinchoninic Acid (BCA) protein concentration determination kit used in the western blotting were obtained from Shanghai Solebao Biotechnology Co., Ltd., whereas the 10% SDS-PAGE reagents were procured from Shanghai Aibisin Biotechnology Co., Ltd., whereas rabbit anti-human N-cadherin, rabbit anti-human vimentin, rabbit anti-human slug, were obtained from Shanghai Aibisin Biotechnology Co., Ltd. Rabbit anti-human β-actin was sourced from Wuhan Elabscience Biotechnology Co., Ltd. The HRP-labeled goat anti-rabbit secondary antibody used was produced by Beijing Zhongshan Jinqiao Biotechnology Co., Ltd., and the ECL luminescence solution used for detection was obtained from Millipore (USA). Both the CCK-8 and apoptosis detection kits were obtained from Shanghai Dongren Chemical Technology Co., Ltd.

### Cell isolation and culture

One gram of endometrial tissue was stripped from the underlying myometrium and dissociated using mechanical and enzymatic digestion as previously described with a few modifications. Briefly, the tissue pieces were washed twice in Phosphate Buffered Saline (PBS) and minced before dissociation in DMEM/F-12 containing 0.1% Bovine Serum Albumin (BSA), 0.5% collagenase I, 40 μg/mL deoxyribonuclease type I (Sigma Aldrich), and 1% penicillin/streptomycin for 40 min at 37 °C in a SI50 Orbital Incubator (Stuart Scientific). The resulting cell solution was filtered through a sterile 70-μm cell strainer (Fisher Scientific) to separate single cells from undigested tissue fragments following isolation. Patient endometrial SCs (EuSCs, EcSCs, and control cells) were then cultured at 37 °C and 5% CO_2_ in DMEM supplemented with 10% heat-inactivated fetal bovine serum. Cells were passaged upon reaching 90% confluence.

### Quantitative real-time PCR (qRT-PCR)

When the endometrial SCs reached logarithmic growth, total RNA was extracted using the TRIzol method (Dalian Bao Biological Engineering Co., Ltd). This RNA was then used as a template in qRT-PCR assays using a qRT-PCR amplification kit from TaKaRa (Dalian Bao Biological Engineering Co., Ltd). The miRNA-223 mimics (sequence 5’-CGTGTATTTGACAAGCTGAGTT-3’) were obtained from TaKaRa (Dalian Bao Biological Engineering Co., Ltd.) and U6 was used as the internal reference for all evaluations. Relative expression was calculated using the 2^−△△Ct^ method.

### Lentivirus infection

Before transduction, the optimal Multiplicity of Infection (MOI) value and related transduction conditions were determined as follows. The cells were digested, suspended, and inoculated into a 24-well plate. When the cells reached approximately 50% confluence, each well was treated with 20 μL of lentivirus and allowed to grow for a further 72 h at 37 °C and 5% CO_2_. Following this, transduced cells were evaluated for green fluorescence using a fluorescence microscope, and fluorescence was calculated as a proportion of the total number of cells in the bright-field images.

### Western blot

sh-NC and sh-miR-223 cells were lysed in RIPA buffer and protein concentration was determined using the BCA method. Samples (20 μg protein) were separated by 10% SDS-PAGE, transferred to PVDF membranes, and blocked with 5% skimmed milk for 1h at room temperature. Membranes were then incubated with the appropriate primary antibodies overnight on a shaker at 4 °C. The primary antibody dilutions were as follows: rabbit anti-N-cadherin (1:1000), rabbit anti-vimentin (1:1000), rabbit anti-Slug (1:1000), rabbit anti-β-actin (1:2000). Membranes were then incubated with the HRP-labeled goat anti-rabbit secondary antibody (1:3000) for 1h at room temperature and then evaluated using an ECL system.

### Wound healing assay

sh-miR-223 and sh-NC cells were also applied to a wound healing assay. Briefly, following digestion and resuspension, the cells were evenly inoculated in 6-well plates and allowed to reach 100% confluence. The authors then created a scratch in the cell monolayer perpendicular to the bottom of the plate using a 10 μL pipette tip. Wells were washed with PBS to remove dislodged cells and 2 mL of serum-free medium was added to each well. The scratch was then observed under an inverted optical microscope and scratch closure was evaluated using Image J software 24 h after initial wounding.

### Transwell assay

To evaluate the migratory potential of the treated SCs, the authors used a Transwell chamber assay. These chambers were pre-coated with Matrigel (serum-free medium: Matrigel = 7:1) and sh-miR-223 and sh-NC cells (5×10^4^ per well) were seeded in the upper chamber using serum-free medium before adding 600 μl of complete medium to the lower chamber. The cells were incubated at 37 °C and 5% CO_2_ for 48 h. Cells in the upper compartment were wiped with a cotton swab and the cells in the bottom chamber were fixed, stained, and evaluated using an optical microscope. Three random high-magnification fields were imaged, and the cells were counted.

### Cell Counting Kit-8 (CCK8) proliferation assay

sh-miR-223 and sh-NC cells were grown to logarithmic phase and then placed in suspension. Cell concentrations were then adjusted to 5×10^4^ cells/mL and inoculated in a 96-well plate at 100 μL per well and cultured for 1, 3, 5, or 7 days. The original medium was then changed to 100 μL of medium and 10 μL CCK-8 reagent per well, and the plates were then incubated at 37 °C for 2 h. The absorbance at 450 nm was then determined for each well.

### Apoptosis assays

Cells were digested, collected, and centrifuged, before being washed in PBS twice and then resuspended in 1× binding buffer at an adjusted cell density of 1×10^6^ cells/mL. A total of 100 μL of these cell suspensions (1×10^5^ cells) was added to each flow tube and then treated with 5 μL FITC-annexin V and 10 μL Propidium Iodide (PI). The cells were gently mixed and incubated in the dark at room temperature for 15 min before adding another 400 μL of 1× binding buffer. The cells were then left for 1 h before being evaluated for apoptosis using a flow cytometer.

### Statistical analysis

GraphPad Prism 8.0 software was used for all statistical analyses. The data are expressed as the mean ± SD of at least three independent experiments and the Student's *t*-test was used to compare values in the sh-miRNA-223 and sh-NC groups; p < 0.05 was considered statistically significant.

## Results

### Expression of miRNA-223 in endometrial SCs

**Fig. 1:**
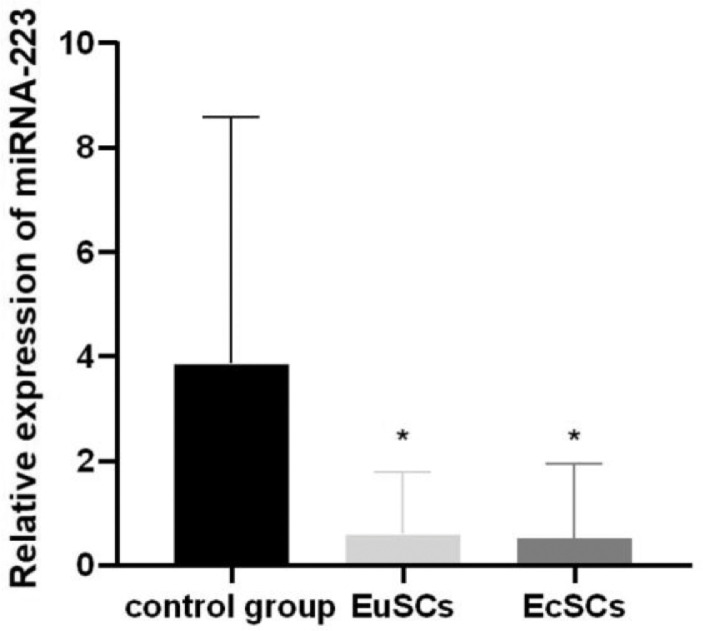
Relative expression of miRNA-223 in control cells, EuSCs, and EcSCs. Expression level of miRNA-223 in EuSCs and EcSCs were significantly lower than those in the control group. miRNA-223, microRNA-223; EuSCs, eutopic endometrial stromal cells; EcSCs, ectopic endometrial stromal cells; *p < 0.05, when compared with the control cells.

The expression of miRNA-223 in patient-derived endometrial SCs (EuSC and EcSC group) was significantly lower than that in the control group (normal endometrial eutopic SCs) (p < 0.05). There was no significant difference in miRNA-223 expression between EuSCs and EcSCs derived from endometriosis patients (Fig. 1).

### Efficiency of the lentiviral transductions

**Fig. 2:**
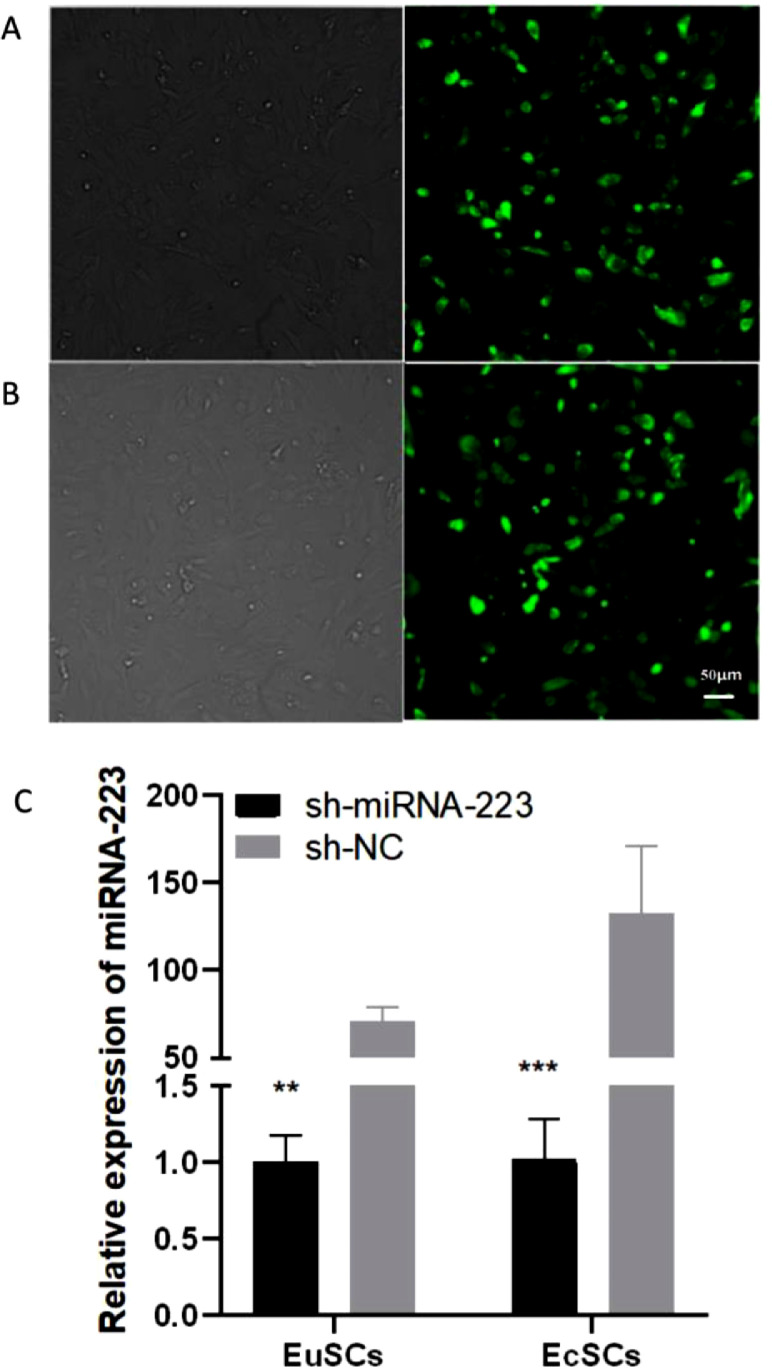
Efficiency of the lentiviral transductions. (A-B) sh-miR-223 and sh-NC green fluorescent protein and miRNA-223 expression in EuSCs (A) and EcSCs (B); (C) miRNA-223 expression in cells transfected with sh-miR-223 or sh-NC was detected using qRT-PCR. Left panels show the light microscope images from each treatment, and the right panels show the fluorescence microscopy images of the same cells. miRNA-223, microRNA-223; EuSCs, eutopic endometrial stromal cells; EcSCs, ectopic endometrial stromal cells; *p < 0.05, when compared to the sh-NC group.

EuSCs and EcSCs were transduced using lentiviral vectors. Transduction efficiency was observed using a fluorescence microscope, and Green Fluorescent Protein (GFP) expression was evaluated. Both light and fluorescence microscopy were used to determine the efficacy of these infections (Fig. 2A). Optimal conditions produced a final transduction efficiency of > 90% and qRT-PCR results confirmed a significant upregulation in miRNA-223 expression in the sh-miR-223 group when compared to that in the sh-NC group (Fig. 2B).

### Expression of the EMT-related proteins PARP-1 and HIF-1α following miRNA-223 upregulation in EuSCs and EcSCs

**Fig. 3:**
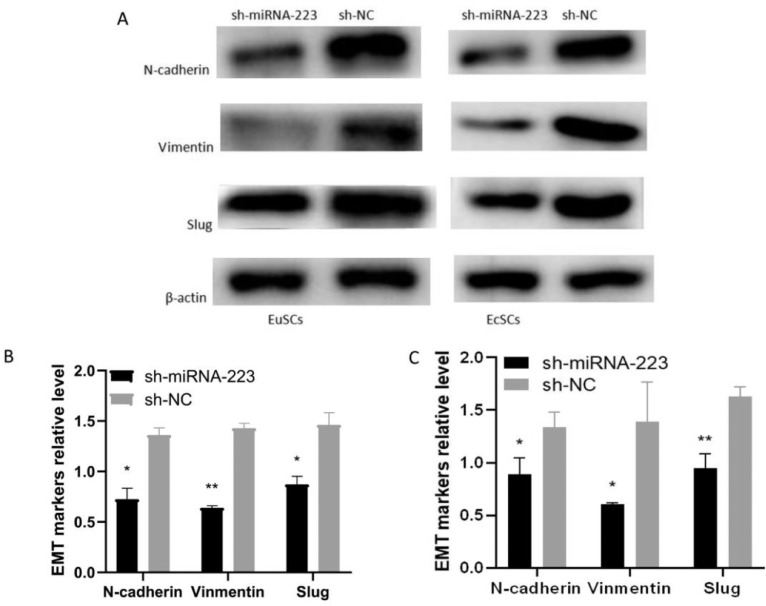
Expression of EMT-related proteins in sh-miR-223- and sh-NC-treated EuSCs and EcSCs (A) Expression of EMT-related proteins in sh-miR-223 and sh-NC cells detected by western blot. (B) Densitometric analysis of protein expression in EuSCs following miRNA-223 upregulation. (C) Densitometric analysis of protein expression in EcSCs. Western blot shows that N-cadherin, vimentin, and Slug expression were all downregulated in response to increased miR-223. miRNA-223, microRNA-223; EuSCs, eutopic endometrial stromal cells; EcSCs, ectopic endometrial stromal cells; *p < 0.05 and **p < 0.01, n ≥ 3, when compared with the sh-NC group.

Western blot revealed that the protein expression of mesenchymal markers N-cadherin, vimentin, and Slug was lower in EuSCs and EcSCs overexpressing miRNA-223 compared to that in the sh-NC control (Fig. 3), suggesting that EMT was inhibited following miRNA-223 upregulation.

### Upregulation of miRNA-223 inhibits cell migration

**Fig. 4:**
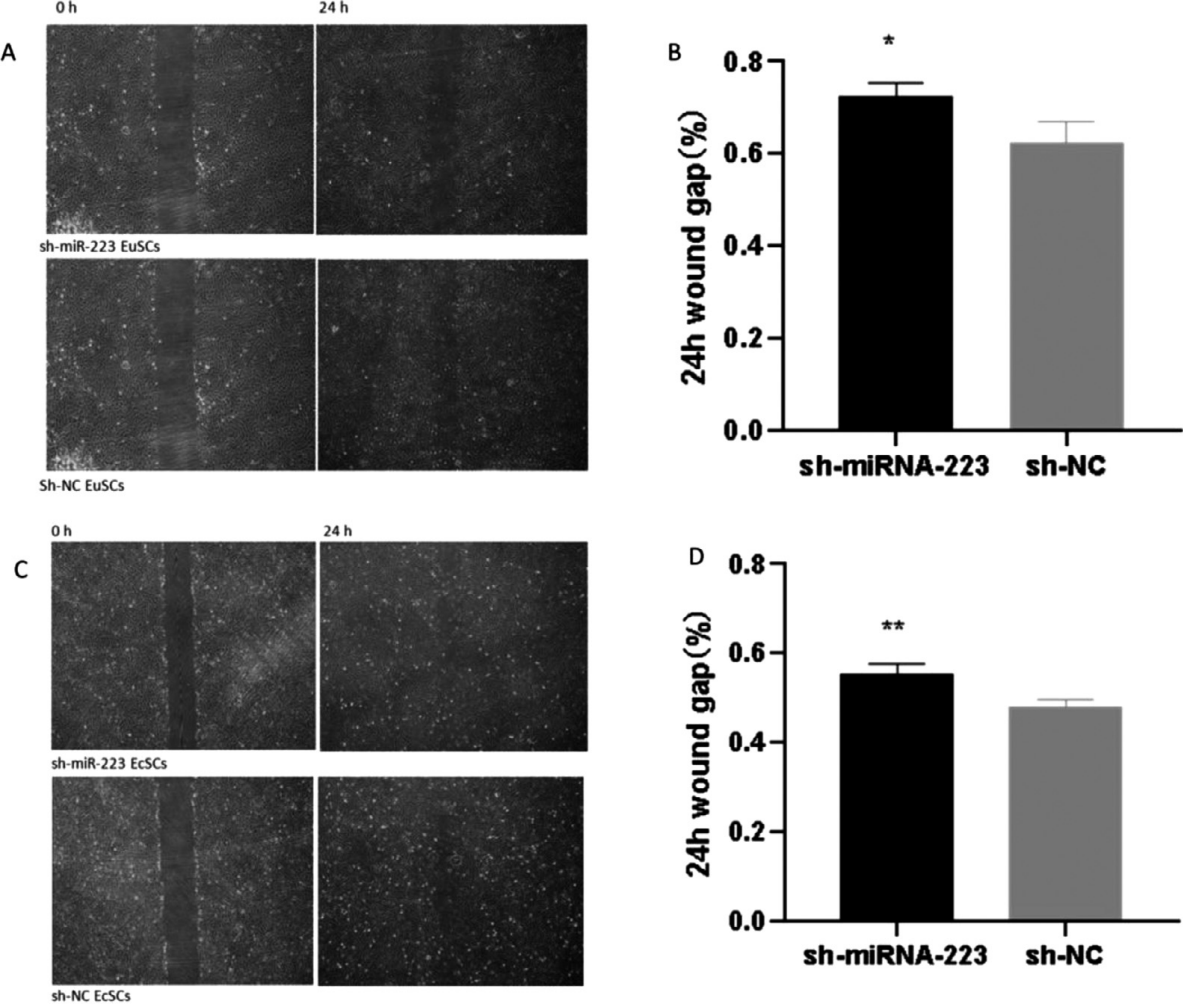
Upregulation of miRNA-223 inhibits cell migration. Wound healing assay demonstrating the changes in cell migration and invasiveness following the addition of sh-miR-223 or sh-NC. (A) Wound healing assay (×200). (B) Quantification of the wound gap in sh-miR-223 and sh-NC EcSCs 24h after scratching. (C) Wound healing assay (×200). (D) Quantification of the wound gap in sh-miR-223 and sh-NC EcSCs 24 h after scratching. miRNA-223, microRNA-223; EuSCs, eutopic endometrial stromal cells; EcSCs, ectopic endometrial stromal cells; *p < 0.05 and **p < 0.01, when compared with the sh-NC group.

Wound healing assay results revealed that sh-miR-223 cells experienced a significantly reduced wound healing ability over a 24 h period in response to a scratch in the monolayer when compared to the control (Fig. 4A and C). This suggests that cell migration was significantly decreased in sh-miR-223 cells when compared to that in sh-NC cells (Fig. 4B and D). Based on these results, the authors can assume that miRNA-223 upregulation reduced the invasion and migration ability of both EuSCs and EcSCs.

### Upregulation of miRNA-223 compromises cellular invasion ability

**Fig. 5:**
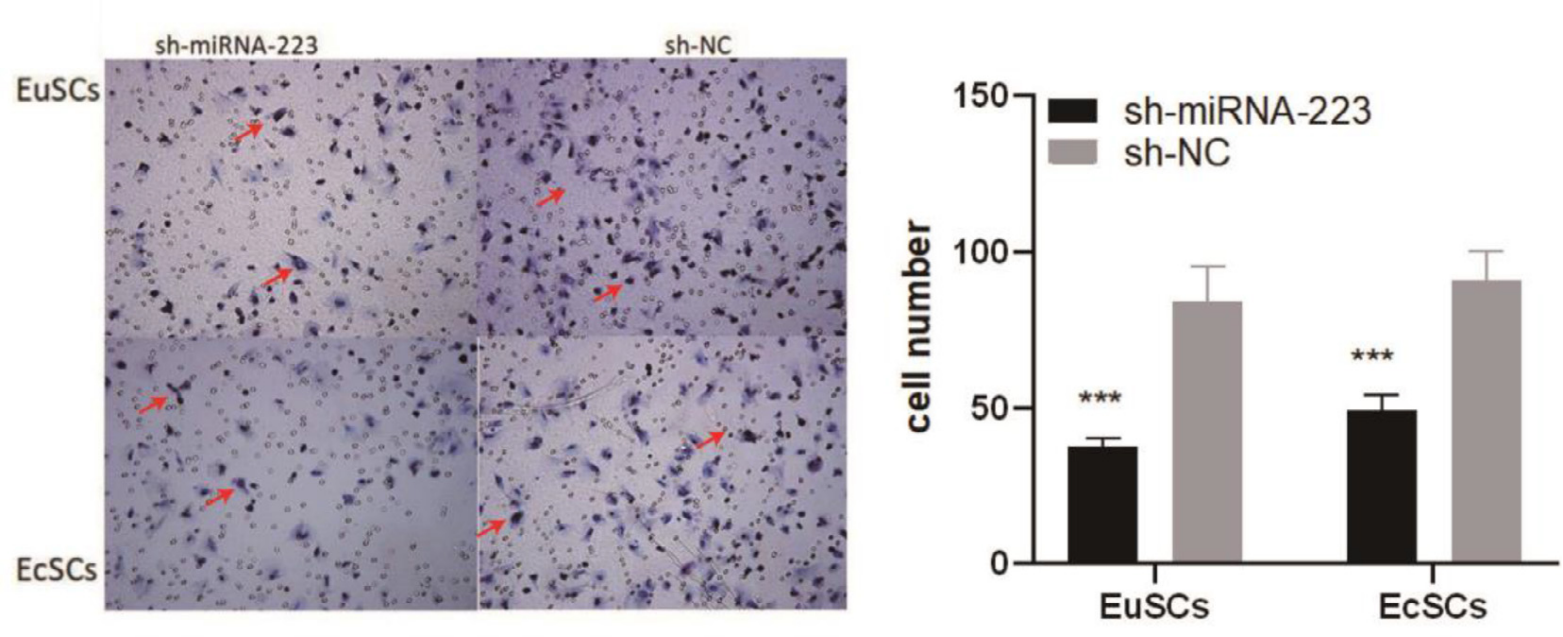
Upregulation of miRNA-223 compromises cellular invasion ability. Transwell assay demonstrates cell invasiveness following treatment with sh-miR-223 or sh-NC. miRNA-223, microRNA-223; EuSCs, eutopic endometrial stromal cells; EcSCs, ectopic endometrial stromal cells; ***p < 0.001, when compared with the sh-NC group.

Transwell assays compared the invasion ability of each of the cell groups. High-magnification imaging of the transwell chamber showed that the number of EuSCs and EcSCs overexpressing miRNA-223 in the lower chamber were significantly reduced compared with those in the sh-NC group, indicating that a decrease in miRNA-223 levels in the endometrial SCs from endometriosis patients reduced the migration and invasion ability of these cells (Fig. 5).

### miRNA-223 upregulation reduces cell proliferation

**Fig. 6:**
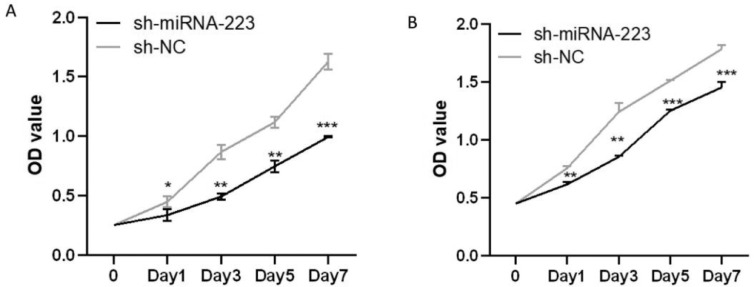
miRNA-223 upregulation reduces cell proliferation. (A) Proliferation of EuSCs in the sh-NC and sh-miRNA-223 groups. (B) Proliferation of EcSCs in the sh-NC and sh-miRNA-223 groups. miRNA-223, microRNA-223; EuSCs, eutopic endometrial stromal cells; EcSCs, ectopic endometrial stromal cells; *p < 0.05, **p < 0.01, and ***p < 0.001, when compared with the sh-NC group.

Proliferation assay results revealed that the proliferation rate of sh-miR-223 cells was lower than that of the sh-NC cells for both EuSCs and EcSCs, indicating that miRNA-223 upregulation may inhibit the proliferation of patient-derived endometrial SCs (Fig. 6).

### Upregulation of miRNA-223 promotes apoptosis

**Fig. 7:**
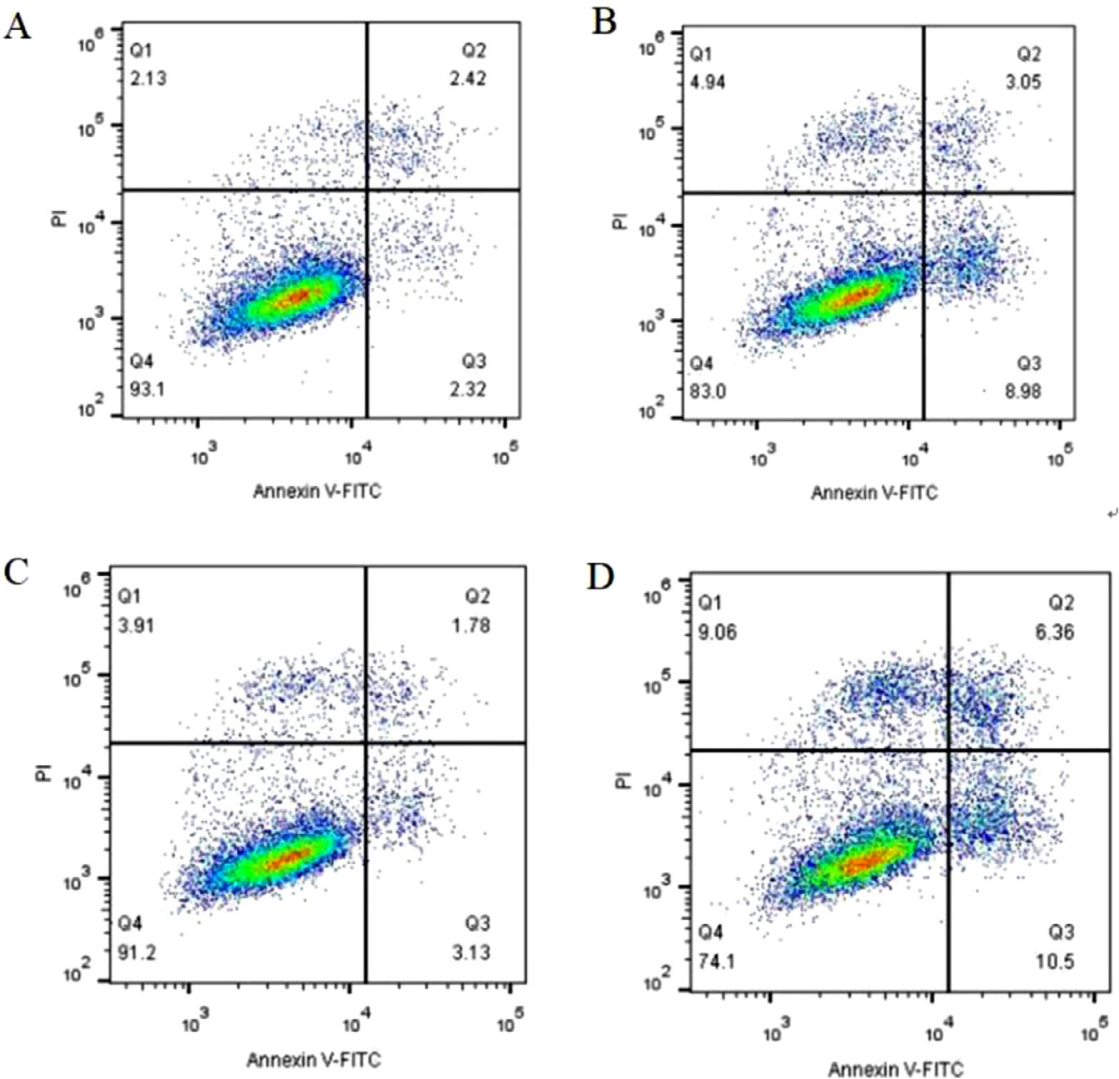
Upregulation of miRNA-223 promotes apoptosis. (A) Apoptosis rate of EuSCs treated with sh-NC. (B) Apoptosis rate of EuSCs treated with sh-miR-223. (C) Apoptosis rate of EcSCs treated with sh-NC. (D) Apoptosis rate of EcSCs treated with sh-miR-223. Q2, indicate the early apoptotic cell; Q3 indicate the late apoptotic cell. miRNA-223, microRNA-223; EuSCs, eutopic endometrial stromal cells; EcSCs, ectopic endometrial stromal cells.

The apoptosis rate of the EuSC sh-miR-223 cells (Fig. 7A) was (12.04% ± 1.84%), whereas that of the sh-NC cells (Fig. 7B) was (4.16% ± 0.84%); this difference was statistically significant (p < 0.01). The apoptosis rate of EcSC sh-miR-223 cells (Fig. 7C) was (15.70% ± 1.25%) and was significantly higher than that of sh-NC cells (Fig. 7D), which was (5.25% ± 0.74%) (p < 0.01). These results indicate that the upregulation of miRNA-223 expression promotes apoptosis in endometrial SCs from endometriosis patients.

## Discussion

While endometriosis is a benign disease, it is characterized by a wide range of pathological changes, including increased invasion and aberrant growth of the endometrial tissues. Further, disease relapse is common. Overall, endometriosis has a serious impact on the bodily functions of patients, severely compromising their daily life. Thus, this condition has become a major focus in obstetrics and gynecological research. However, the pathogenesis of endometriosis is complex and unclear, with the classical theory being that of retrograde menstruation proposed by Sampson.^10^

According to previous studies, miRNA-223 is specifically expressed and involved in a variety of diseases.^4^ In particular, miRNA-223 expression was shown to be upregulated in gastric^11^ and ovarian cancer^12^ and downregulated in the liver^13^ and lung cancer.^14^ miRNA-223 has been reported to play a crucial role in the tumorigenesis, development, and metastasis of a number of malignancies and thus was also suggested as a diagnostic and prognostic biomarker in these pathologies.^15^ This study evaluated the expression of miRNA-223 in endometrial SCs from 26 patients with endometriosis and 14 hysteromyoma patients. miRNA-223 was significantly downregulated in the endometrial SCs of endometriosis patients.

EMT is also involved in the pathogenesis of various diseases. In normal tissues, cells are arranged in tight, ordered patterns; in contrast, tumor cells are loosely connected, and their migration and invasion abilities are greatly enhanced. The activation of EMT-related transcription factors leads to the inhibition of epithelial cell marker expression and an upregulation of SC markers. This often results in the reduced expression of cell adhesion proteins (such as E-cadherin) as well as increased expression of cytokeratin and N-cadherin, which reduce cell adhesion,^16^^,^^17^ improving the cell migration ability.

Vimentin is a structural protein that promotes cell migration^18^ and Slug is a zinc-finger protein that regulates EMT mainly by suppressing E-cadherin.^19^ Overall, EMT enhances the ability of cells to migrate and invade tissues.^20^ Studies have shown that miRNA-223 is involved in the regulation of EMT in certain diseases. The present study's results reveal that the EMT-related molecules N-cadherin, vimentin, and Slug were suppressed in both EuSCs and EcSCs when the miRNA-223 expression was upregulated. These observations indicate that miRNA-223 reversed EMT in SCs from endometriosis tissues. Transwell and wound healing experiments confirmed that the migration and invasion abilities of these cells were significantly reduced, indicating that miRNA-223 regulates EMT in endometriosis. The specific mechanism underlying miRNA-223-mediated EMT regulation remains unclear. A previous study, in nasopharyngeal carcinoma, reported that miRNA-223 functions as a tumor suppressor, and its effects were primarily mediated via the downregulation of SSRP1 and inhibition of EMT.^21^ FBW7 was previously identified as a functional target of miRNA-223 in non-small cell lung cancer cells, suggesting a critical role for the miR-223/FBW7 pathway in regulating EMT and chemosensitivity.^22^

In this study, the authors found that the upregulation of miRNA-223 expression in EuSCs and EcSCs resulted in reduced cellular proliferation and enhanced apoptosis. The mechanism underlying miRNA-223-mediated regulation of cellular proliferation and apoptosis is complex. In colorectal cancer, downregulating miR-223 expression enhanced FoxO3a and BIM expression, suppressing SW620 cell proliferation and inducing apoptosis.^23^ In hepatocellular carcinoma, miRNA-223 inhibited tumorigenesis and promoted apoptosis through the mTOR signaling pathway *in vitro*.^24^ In addition, miRNA-223 inhibited proliferation and enhanced apoptosis in acute myeloid leukemia cell lines by directly targeting F-box and WD repeat domain containing 7.^25^

In conclusion, miRNA-223 expression was downregulated in endometrial SCs from endometriosis patients. By upregulating miRNA-223 expression, the expression of EMT-related molecules N-cadherin, vimentin, and Slug was suppressed in both EuSCs and EcSCs. Upregulation of miRNA-223 expression also inhibited endometrial SC proliferation, invasion, and migration, reversing the EMT. Based on these results, it is suggested that miRNA-223 is a potential therapeutic target for endometriosis.

## CRediT authorship contribution statement

**Yuan Xue:** Conceptualization, Data curation, Formal analysis, Methodology. **Xueyan Lin:** Formal analysis, Validation. **Tingting Shi:** Formal analysis, Validation. **Yongjie Tian:** Conceptualization, Data curation, Formal analysis, Funding acquisition, Methodology, Writing – original draft, Writing – review & editing.

## Funding

This work was supported by the Natural Science Foundation of Shandong Province (grant number ZR2019MH129).

## Declaration of Competing Interest

The authors declare that they have no known competing financial interests or personal relationships that could have appeared to influence the work reported in this paper.

## Appendix Supplementary materials

Image, image 1

Image, image 2

Image, image 3

Image, image 4

Image, image 5

Image, image 6

Image, image 7

Image, image 8
