# Supplementary figures and images for: miRNA-223 expression in patient-derived eutopic and ectopic endometrial stromal cells and its effect on epithelial-to-mesenchymal transition in endometriosis
Source: Clinics (Sao Paulo). 2022 Oct 14;77:100112. doi: 10.1016/j.clinsp.2022.100112 (PMC9579502; doi:10.1016/j.clinsp.2022.100112)

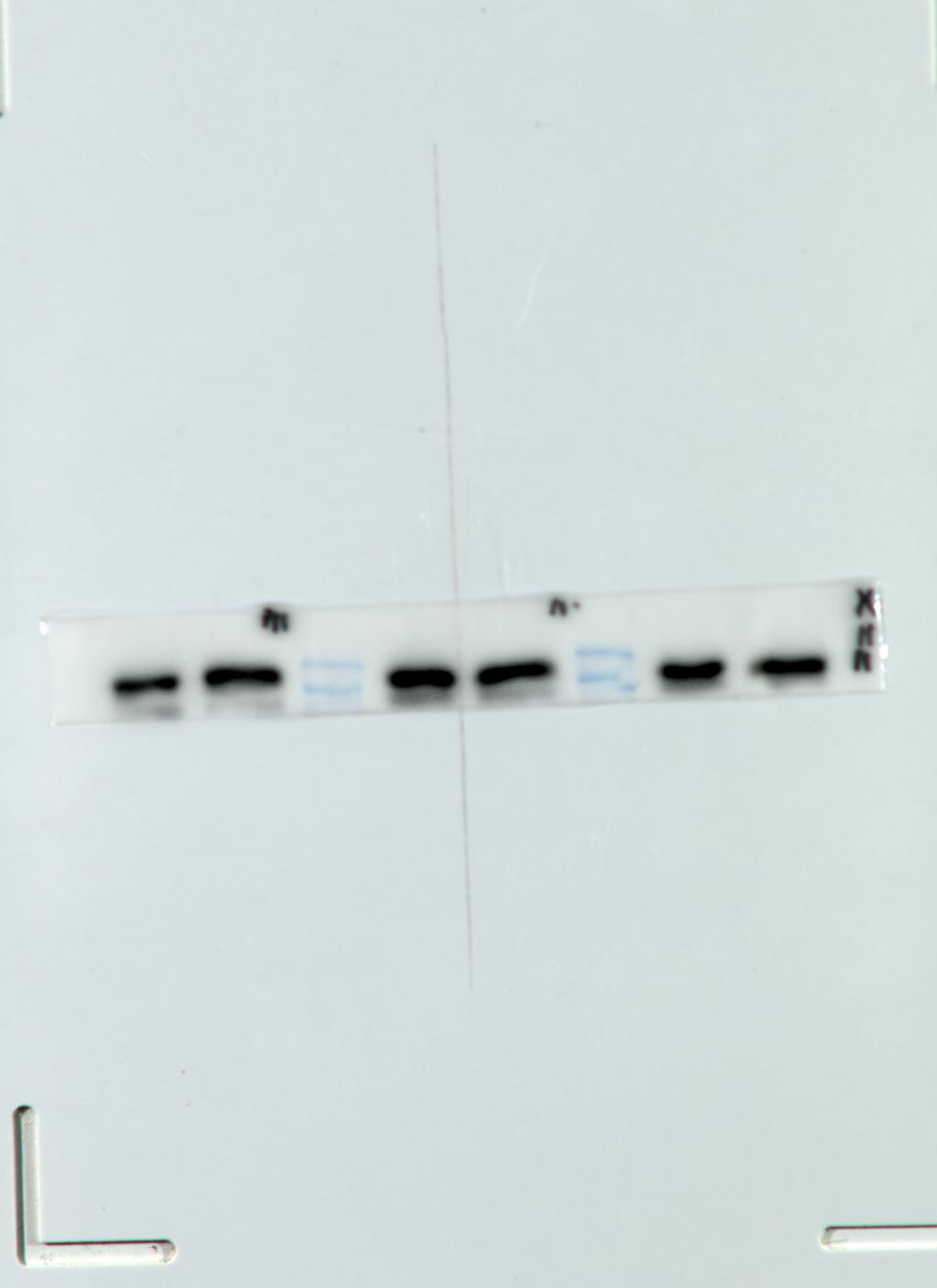

Supplement: Supplementary file 1 [file mmc1.zip › mmc1.jpg]

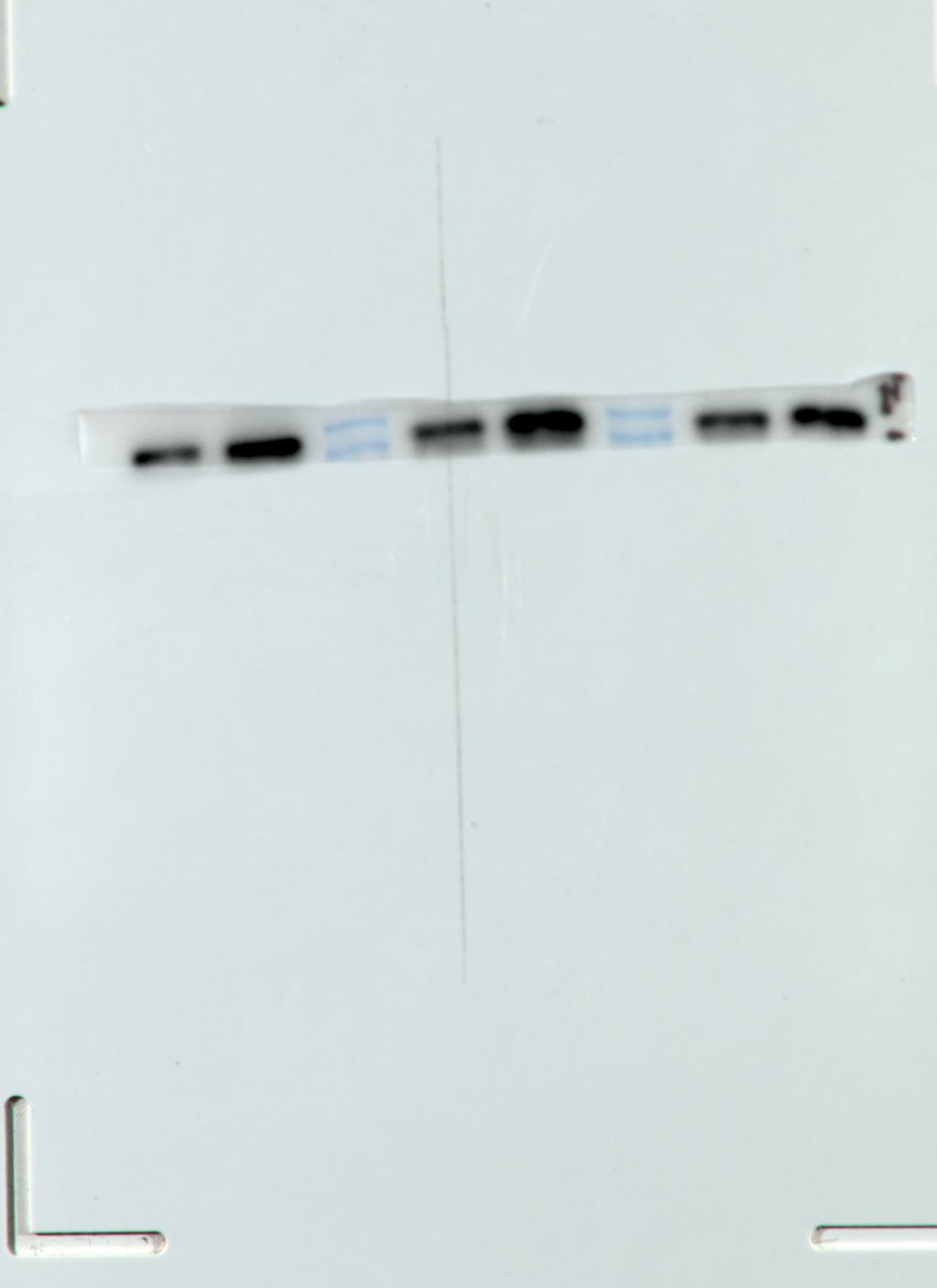

Supplement: Supplementary file 2 [file mmc2.zip › mmc2.jpg]

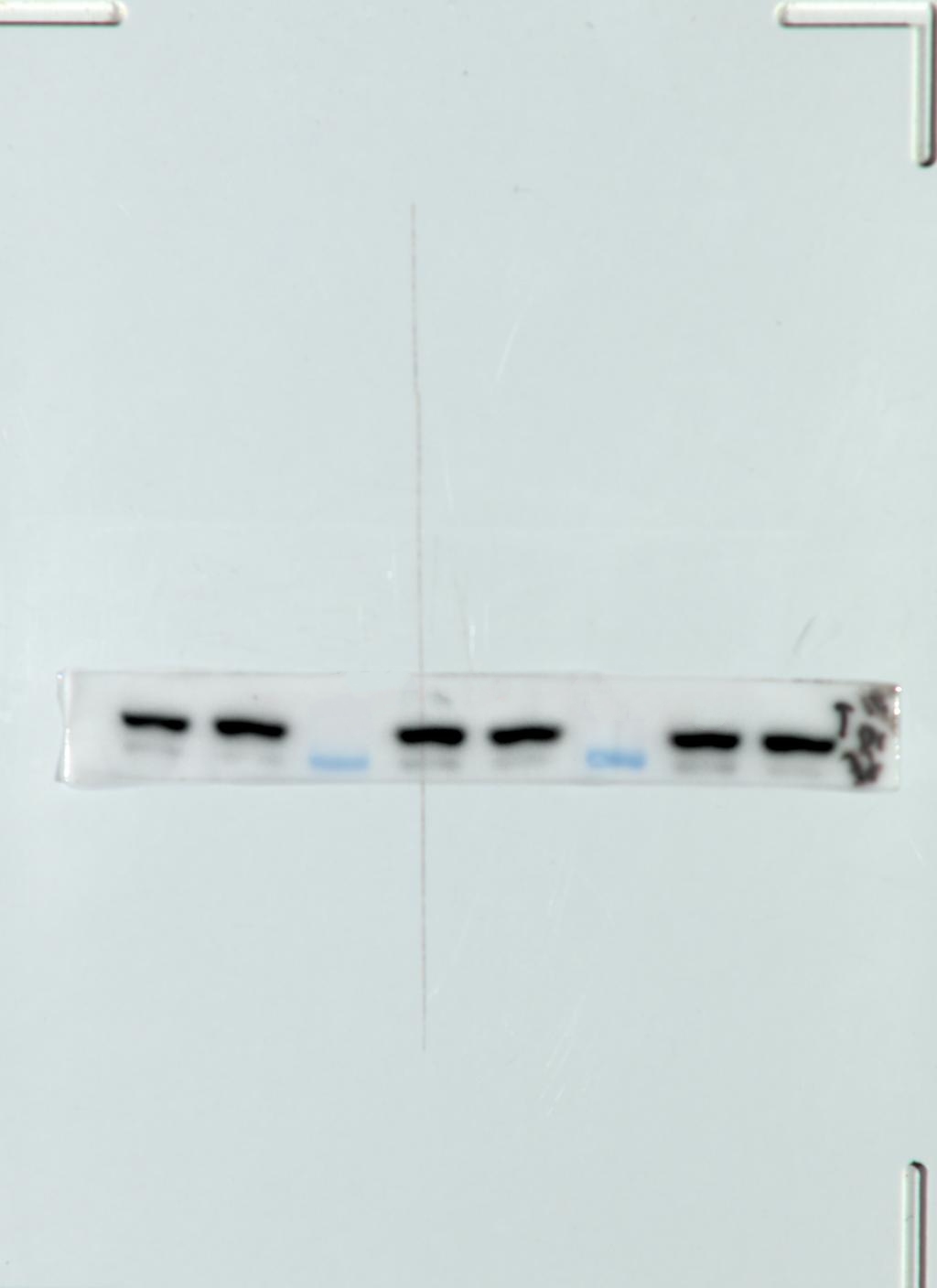

Supplement: Supplementary file 3 [file mmc3.zip › mmc3.jpg]

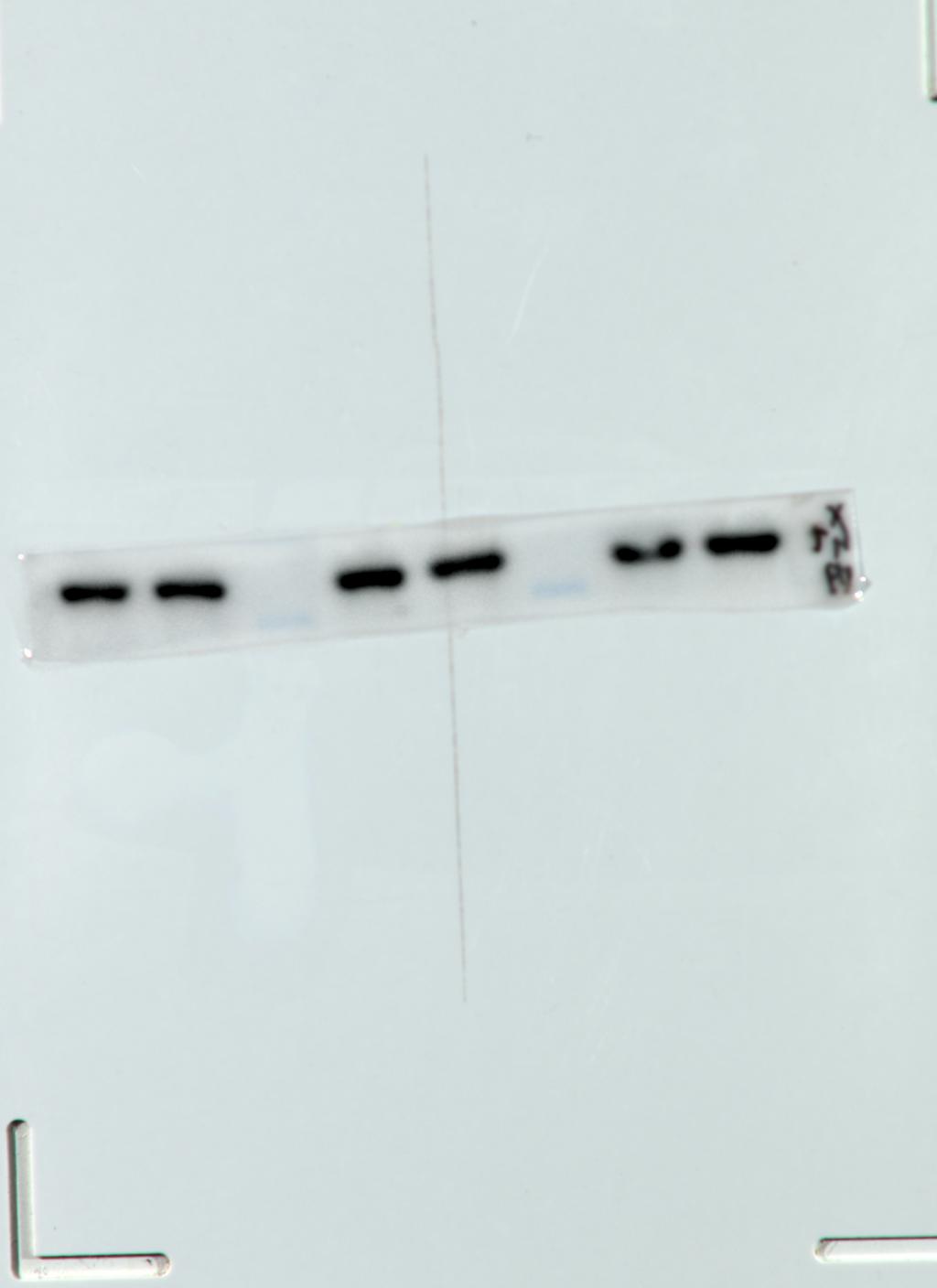

Supplement: Supplementary file 4 [file mmc4.zip › mmc4.jpg]

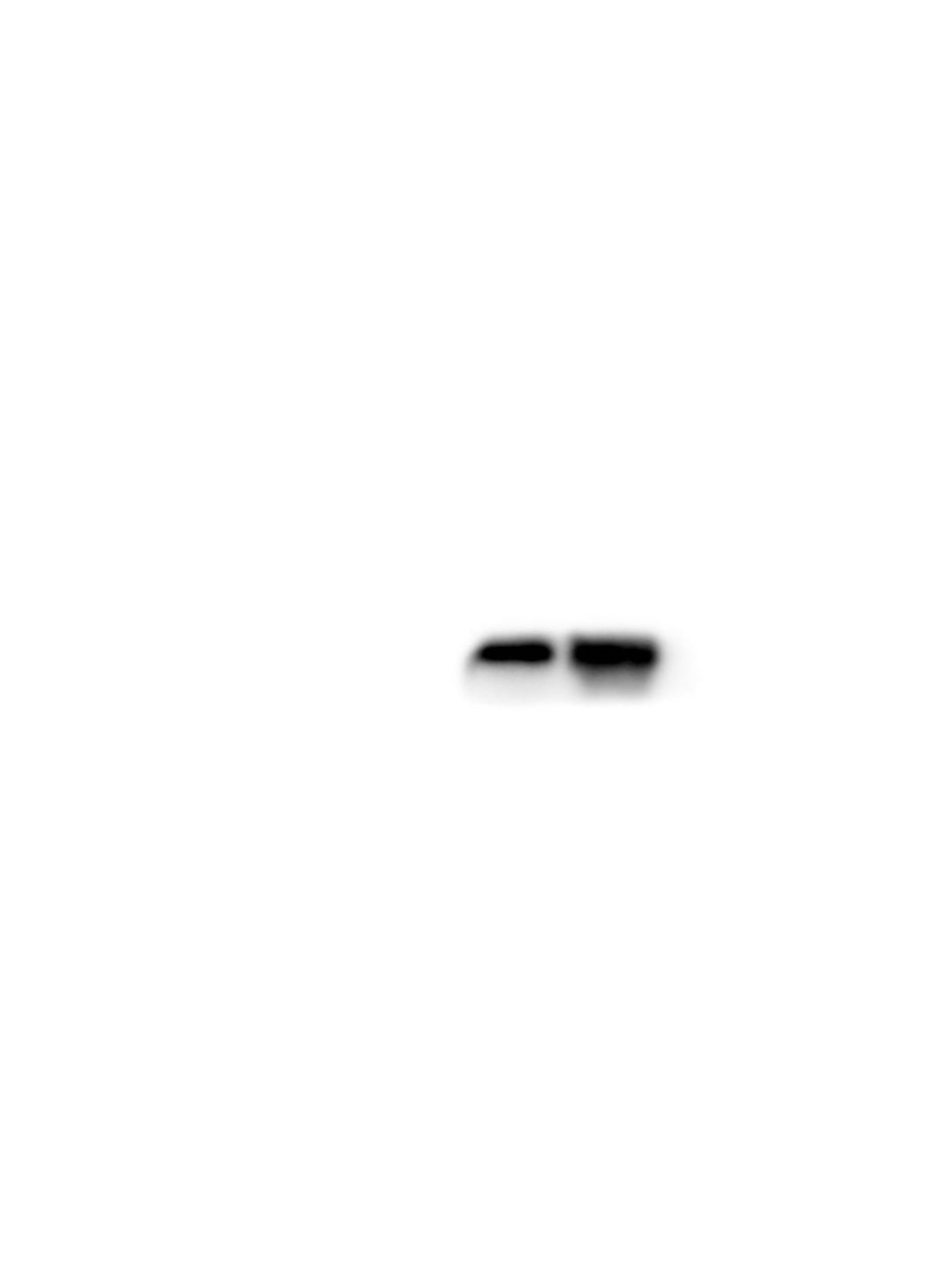

Supplement: Supplementary file 5 [file mmc5.zip › mmc5.jpg]

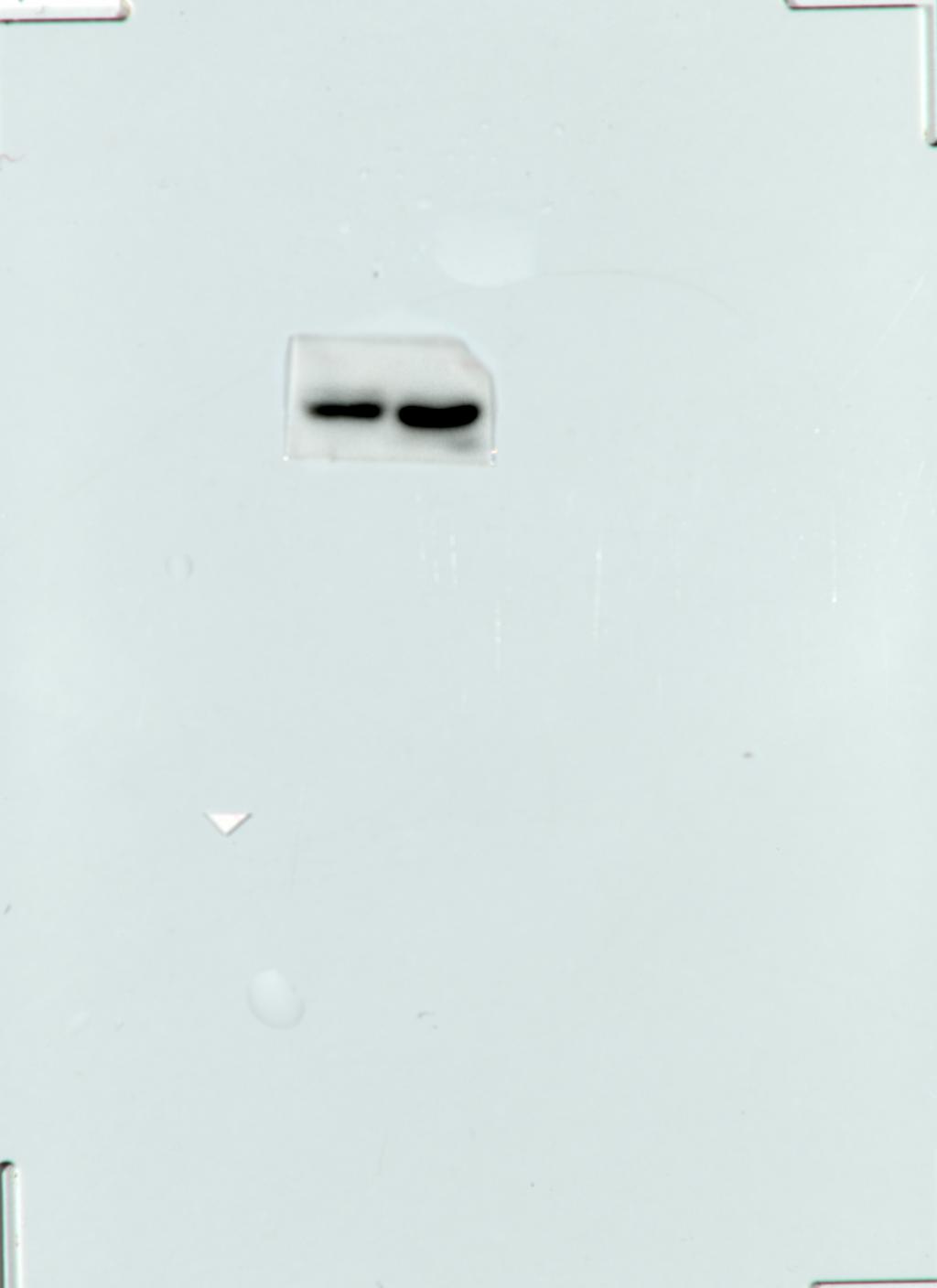

Supplement: Supplementary file 6 [file mmc6.zip › mmc6.jpg]

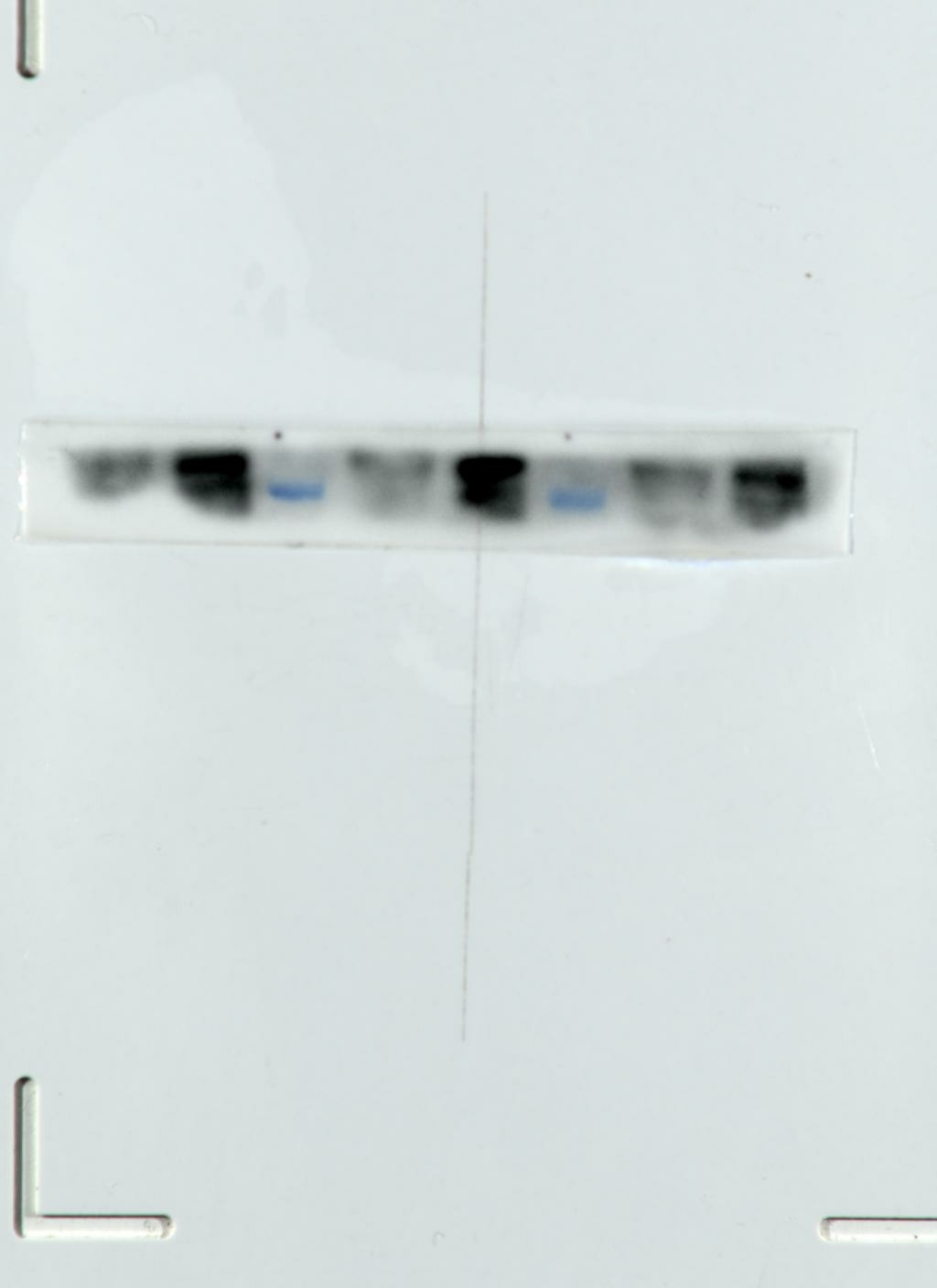

Supplement: Supplementary file 7 [file mmc7.zip › mmc7.jpg]

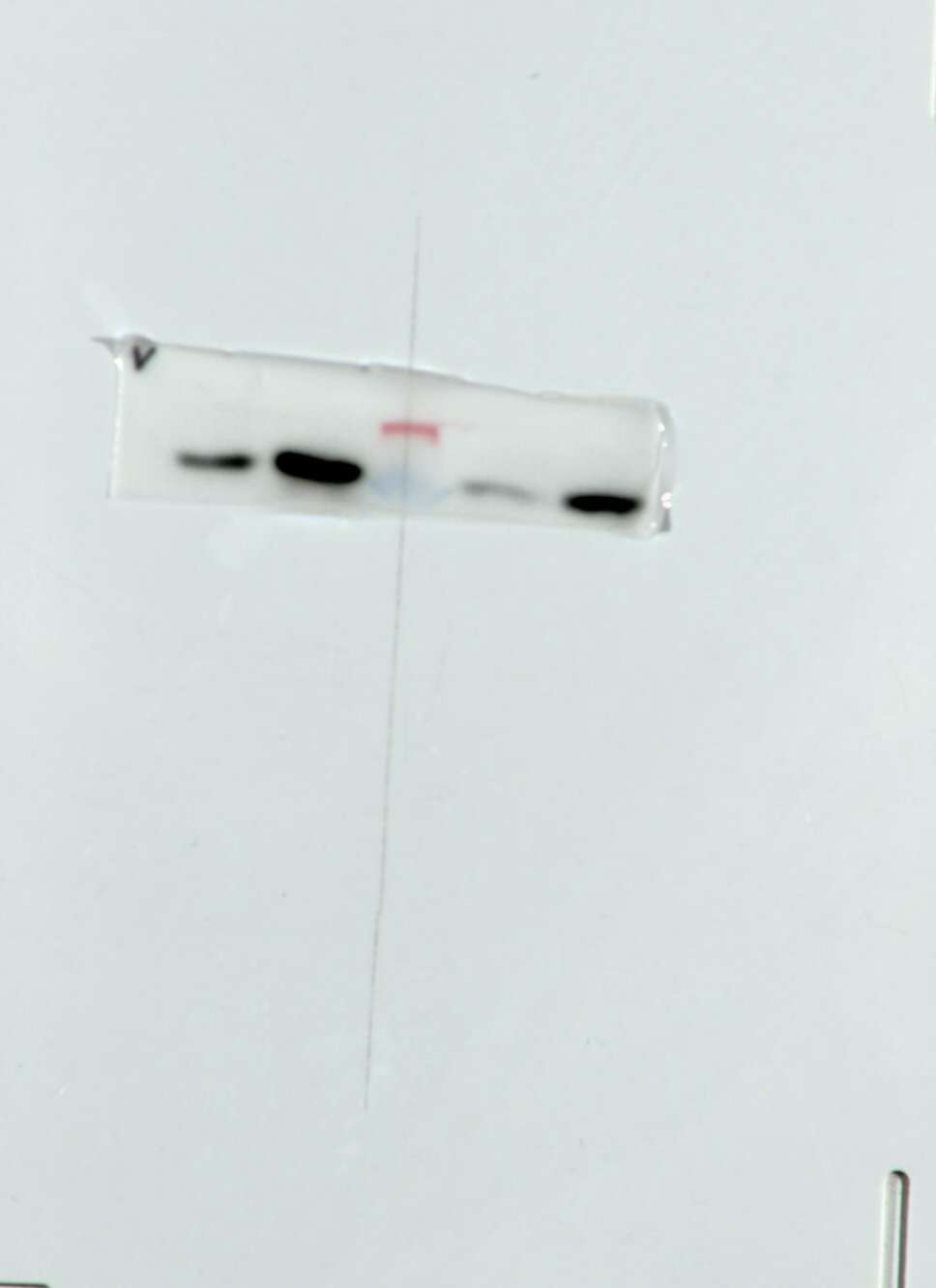

Supplement: Supplementary file 8 [file mmc8.zip › mmc8.jpg]
